# Penta­carbonyl-2κ*^5^C-*chlorido-1κ*Cl-*bis­[1(η^5^)-cyclo­penta­dien­yl][μ_2_-oxido(meth­yl)methyl­ene-1:2κ^2^ *O*:*C*]tungsten(0)zirconium(IV)

**DOI:** 10.1107/S1600536808028006

**Published:** 2008-09-06

**Authors:** Catharine Esterhuysen, Arno Neveling, Nyambeni Luruli, Gert J. Kruger, Stephanie Cronje

**Affiliations:** a Department of Chemistry and Polymer Science, University of Stellenbosch, Private Bag X1, Matieland 7602, South Africa; b Department of Chemistry and Biochemistry, University of Johannesburg, PO Box 524, Auckland Park, Johannesburg 2006, South Africa

## Abstract

The title compound, [ZrW(C_5_H_5_)_2_(C_2_H_3_O)Cl(CO)_5_] or [W(CO)_5_C(CH_3_)OZr(C_5_H_5_)_2_Cl], consists of two metal centres, with a (tungsten penta­carbon­yl)oxymethyl­carbene group coordinating as a monodentate ligand to the chloridozirconocene. The two halves of the mol­ecule are related by a crystallographic mirror plane. Delocalization through the Zr—O—C=W unit is indicated by a short Zr—O distance and a nearly linear Zr—O—C angle.

## Related literature

For related literature regarding catalytic data of the title compound, see: Sinn *et al.* (1980 ▶); Brüll *et al.* (2001 ▶); Luruli *et al.* (2004 ▶, 2006 ▶). For comparable structures, see: Erker *et al.* (1989 ▶); Wolczanski *et al.* (1983 ▶); Esterhuysen *et al.* (2008 ▶). For a description of the Cambridge Structural Database, see: Allen (2002 ▶).
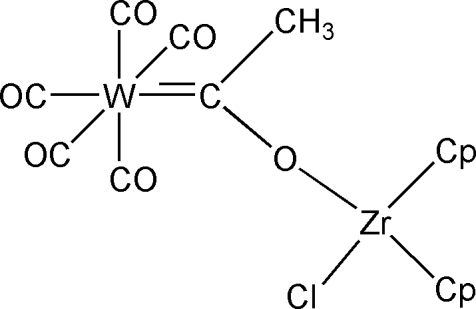


## Experimental

### 

#### Crystal data

[ZrW(C_5_H_5_)_2_(C_2_H_3_O)Cl(CO)_5_]
*M* *_r_* = 623.79
Orthorhombic, 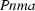

*a* = 22.3794 (8) Å
*b* = 12.3852 (7) Å
*c* = 7.2404 (3) Å
*V* = 2006.85 (16) Å^3^
*Z* = 4
Mo *K*α radiation
μ = 6.41 mm^−1^
*T* = 273 (2) K
0.34 × 0.31 × 0.29 mm

#### Data collection

Philips PW1100 diffractometer
Absorption correction: ψ scan (North *et al.*, 1968 ▶) *T*_min_ = 0.219, *T*_max_ = 0.258 (expected range = 0.133–0.156)
2070 measured reflections
1851 independent reflections
1521 reflections with *I* > 2σ(*I*)
*R*_int_ = 0.042
3 standard reflections every 50 reflections intensity decay: none

#### Refinement

*R*[*F* ^2^ > 2σ(*F* ^2^)] = 0.032
*wR*(*F* ^2^) = 0.092
*S* = 1.08
1851 reflections
131 parameters
H-atom parameters constrained
Δρ_max_ = 1.15 e Å^−3^
Δρ_min_ = −1.14 e Å^−3^

### 

Data collection: *PWPC* (Gomm, 1998 ▶); cell refinement: *PWPC*; data reduction: *Xtal3*.4 (Hall *et al.*, 1995 ▶); program(s) used to solve structure: *SHELXS97* (Sheldrick, 2008 ▶); program(s) used to refine structure: *SHELXL97* (Sheldrick, 2008 ▶); molecular graphics: *X-SEED* (Barbour, 2001 ▶; Atwood & Barbour, 2003 ▶); software used to prepare material for publication: *publCIF* (Westrip, 2008 ▶).

## Supplementary Material

Crystal structure: contains datablocks I, global. DOI: 10.1107/S1600536808028006/om2260sup1.cif

Structure factors: contains datablocks I. DOI: 10.1107/S1600536808028006/om2260Isup2.hkl

Additional supplementary materials:  crystallographic information; 3D view; checkCIF report

## Figures and Tables

**Table:** 

W1—C4	2.192 (9)
O4—C4	1.270 (10)
Zr1—O4	2.026 (6)

**Table:** 

C4—O4—Zr1	177.4 (7)

**Fig. 1.**
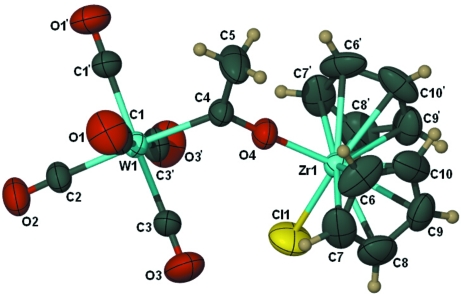
The molecular structure of (II) showing the atomic labelling scheme and displacement ellipsoids drawn at the 50% probability level.

## supplementary crystallographic information

### Comment

Homogeneous equivalents of the heterogeneous catalysts used in Ziegler–Natta polymerization of alkenes are of interest in efforts to understand the mechanism of polymerization. Cp_2_TiCl_2_ (I) has been shown to polymerize ethylene when activated by MAO (Sinn *et al.*, 1980). In our ongoing studies into finding improved catalysts for the oligomerization of α-olefins we have studied zirconocene equivalents to (I) where we replaced one of the Cl ligands with a number of different ligands (Brüll *et al.*, 2001). In particular, the use of a tungsten–carbene moiety as a ligand, (II), has been proven to result in an effective catalyst for the oligomerization of 1-pentene, as well as the copolymerization of ethene and 1-pentene, in the presence of MAO (Luruli *et al.*, 2004; Luruli *et al.*, 2006). Herein we report the crystal structure of the title zirconocene complex, (II).

In the molecular structure the Zr—O distance is shorter than all other zirconocene complexes containing a Zr—O—C(*R*)═*M* (where *M* = any transition metal) group reported to date (Cambridge Structural Database, v. 5.29; Allen, 2002), except when *R* = H [1.971 (4) Å; Wolczanski *et al.*, 1983]. The Zr—O—C angle, on the other hand, is more linear than the previously published structures, with a larger value than that of the benzoxycarbene W(CO)_5_C(C_6_H_5_)OZr(C_5_H_5_)_2_OC_6_H_5_ (166.1 (5)°; Erker *et al.*, 1989). This, together with the short [1.27 (1)Å] C(carbene)—O distance, suggests that the bridging group forms an acyl-type structure, W—C(Me)═O, with a typical hard–hard bond involving σ and π-donation from the O-atom to the Zr-fragment. This is similar to the hafnocene complex W(CO)_5_C(C_6_H_5_)OHf(C_5_H_5_)_2_Cl (Esterhuysen *et al.*, 2008), where the Hf—O—C angle is also nearly linear [171.4 (3)°].

No intermolecular interactions are observed in the crystal structure, with the molecules packing in columns parallel to the *a* axis.

### Experimental

To a well stirred suspension of W(CO)_6_ (8.906 g) in 80 ml diethylether a solution of LiCH_3_ (17 ml, 1.6*M* in diethylether) in 50 ml diethylether was added. After solvent removal, dissolution of the residue in 100 ml cold water and filtration, a solution of Et_4_NCl (8.306 g) in 25 ml cold water was added to the filtrate. Upon filtration 1.015 g of the product {[W(CO)_5_C(CH_3_)O][NEt_4_]} was dissolved in 30 ml dichloromethane and added to a solution of Cp_2_ZrCl_2_ (0.585 g) in 70 ml dichloromethane. After stirring for 30 min at -40°C AgBF_4_ (0.389 g) was added and stirred for 90 min at -40°C. After reaching room temperature the solvent was removed and the residue extracted in 5 portions of 10 ml toluene. The extract was filtered, and the filtrate dried over anhydrous MgSO_4_. The solution was layered with pentane to yield red crystals suitable for X-ray diffraction analysis.

### Refinement

H atoms were positioned geometrically, with C—H = 0.95–0.98 Å, and constrained to ride on their parent atoms, with *U*_iso_(H) = 1.2–1.5*U*_eq_(C).

### Crystal data

**Table:**

[ZrW(C_5_H_5_)_2_(C_2_H_3_O)Cl(CO)_5_]	*D*_x_ = 2.065 Mg m^−^^3^
*M**_r_* = 623.79	Mo *K*α radiation, λ = 0.71073 Å
Orthorhombic, *P**n**m**a*	Cell parameters from 25 reflections
*a* = 22.3794 (8) Å	θ = 2–17°
*b* = 12.3852 (7) Å	µ = 6.41 mm^−^^1^
*c* = 7.2404 (3) Å	*T* = 273 K
*V* = 2006.85 (16) Å^3^	Prism, red
*Z* = 4	0.34 × 0.31 × 0.29 mm
*F*(000) = 1176	

### Data collection

**Table:**

Philips PW1100 diffractometer	1521 reflections with *I* > 2σ(*I*)
Radiation source: fine-focus sealed tube	*R*_int_ = 0.042
graphite	θ_max_ = 25.0°, θ_min_ = 2.5°
ω/2θ scans	*h* = 0→26
Absorption correction: ψ scan (North *et al.*, 1968)	*k* = 0→14
*T*_min_ = 0.219, *T*_max_ = 0.258	*l* = −8→0
2070 measured reflections	3 standard reflections every 50 reflections
1851 independent reflections	intensity decay: none

### Refinement

**Table:**

Refinement on *F*^2^	Primary atom site location: structure-invariant direct methods
Least-squares matrix: full	Secondary atom site location: difference Fourier map
*R*[*F*^2^ > 2σ(*F*^2^)] = 0.032	Hydrogen site location: inferred from neighbouring sites
*wR*(*F*^2^) = 0.092	H-atom parameters constrained
*S* = 1.08	*w* = 1/[σ^2^(*F*_o_^2^) + (0.053*P*)^2^ + 1.7971*P*] where *P* = (*F*_o_^2^ + 2*F*_c_^2^)/3
1851 reflections	(Δ/σ)_max_ = 0.001
131 parameters	Δρ_max_ = 1.15 e Å^−^^3^
0 restraints	Δρ_min_ = −1.14 e Å^−^^3^

### Special details

**Table:**

Geometry. All e.s.d.'s (except the e.s.d. in the dihedral angle between two l.s. planes) are estimated using the full covariance matrix. The cell e.s.d.'s are taken into account individually in the estimation of e.s.d.'s in distances, angles and torsion angles; correlations between e.s.d.'s in cell parameters are only used when they are defined by crystal symmetry. An approximate (isotropic) treatment of cell e.s.d.'s is used for estimating e.s.d.'s involving l.s. planes.
Refinement. Refinement of *F*^2^ against ALL reflections. The weighted *R*-factor *wR* and goodness of fit *S* are based on *F*^2^, conventional *R*-factors *R* are based on *F*, with *F* set to zero for negative *F*^2^. The threshold expression of *F*^2^ > σ(*F*^2^) is used only for calculating *R*-factors(gt) *etc*. and is not relevant to the choice of reflections for refinement. *R*-factors based on *F*^2^ are statistically about twice as large as those based on *F*, and *R*- factors based on ALL data will be even larger.

### Fractional atomic coordinates and isotropic or equivalent isotropic displacement parameters (Å^2^)

**Table:**

	*x*	*y*	*z*	*U*_iso_*/*U*_eq_	Occ. (<1)
W1	0.812162 (16)	0.2500	0.32704 (5)	0.04133 (16)	
Cl1	0.60410 (18)	0.2500	0.5194 (4)	0.0896 (10)	
O1	0.8874 (3)	0.0780 (5)	0.1035 (9)	0.092 (2)	
C1	0.8601 (3)	0.1384 (6)	0.1848 (10)	0.0573 (18)	
Zr1	0.59893 (4)	0.2500	0.18263 (11)	0.0388 (2)	
O2	0.9032 (3)	0.2500	0.6604 (11)	0.078 (2)	
C2	0.8712 (4)	0.2500	0.5381 (14)	0.053 (2)	
O3	0.7386 (3)	0.0625 (5)	0.5187 (10)	0.094 (2)	
C3	0.7643 (3)	0.1307 (6)	0.4506 (10)	0.0529 (17)	
O4	0.6885 (3)	0.2500	0.1422 (10)	0.0535 (17)	
C4	0.7442 (4)	0.2500	0.1090 (13)	0.045 (2)	
C5	0.7572 (6)	0.2500	−0.0914 (16)	0.089 (5)	
H5A	0.8006	0.2500	−0.1105	0.134*	
H5B	0.7399	0.3146	−0.1482	0.134*	0.50
H5C	0.7399	0.1854	−0.1482	0.134*	0.50
C6	0.6081 (5)	0.0817 (7)	−0.0048 (16)	0.088 (3)	
H6	0.6406	0.0757	−0.0886	0.106*	
C7	0.6081 (5)	0.0484 (6)	0.1785 (16)	0.085 (3)	
H7	0.6407	0.0169	0.2432	0.102*	
C8	0.5524 (5)	0.0696 (8)	0.2486 (16)	0.083 (3)	
H8	0.5392	0.0535	0.3703	0.100*	
C9	0.5185 (4)	0.1185 (7)	0.1117 (15)	0.074 (2)	
H9	0.4785	0.1430	0.1246	0.088*	
C10	0.5523 (5)	0.1253 (7)	−0.0444 (13)	0.079 (3)	
H11	0.5399	0.1546	−0.1595	0.095*	

### Atomic displacement parameters (Å^2^)

**Table:**

	*U*^11^	*U*^22^	*U*^33^	*U*^12^	*U*^13^	*U*^23^
W1	0.0383 (2)	0.0444 (2)	0.0413 (2)	0.000	0.00042 (16)	0.000
Cl1	0.118 (3)	0.107 (2)	0.0435 (15)	0.000	0.0088 (17)	0.000
O1	0.099 (4)	0.091 (4)	0.087 (4)	0.026 (4)	0.009 (4)	−0.027 (4)
C1	0.059 (4)	0.058 (4)	0.055 (4)	0.010 (4)	−0.003 (3)	−0.011 (4)
Zr1	0.0376 (5)	0.0378 (5)	0.0408 (5)	0.000	0.0006 (4)	0.000
O2	0.056 (5)	0.109 (7)	0.068 (5)	0.000	−0.016 (4)	0.000
C2	0.044 (5)	0.069 (6)	0.047 (5)	0.000	−0.007 (5)	0.000
O3	0.089 (4)	0.083 (4)	0.111 (5)	−0.019 (4)	0.009 (4)	0.034 (4)
C3	0.051 (4)	0.050 (4)	0.057 (4)	−0.006 (3)	−0.007 (3)	0.014 (3)
O4	0.039 (4)	0.066 (5)	0.055 (4)	0.000	−0.002 (3)	0.000
C4	0.037 (5)	0.050 (5)	0.046 (5)	0.000	0.000 (4)	0.000
C5	0.061 (7)	0.162 (14)	0.045 (6)	0.000	0.000 (6)	0.000
C6	0.096 (7)	0.052 (5)	0.117 (8)	−0.019 (5)	0.039 (7)	−0.040 (6)
C7	0.092 (7)	0.033 (4)	0.130 (10)	0.004 (4)	−0.036 (7)	0.004 (5)
C8	0.104 (7)	0.055 (5)	0.091 (6)	−0.026 (5)	−0.002 (7)	0.015 (5)
C9	0.051 (4)	0.058 (5)	0.112 (7)	−0.013 (4)	−0.013 (5)	−0.002 (5)
C10	0.123 (8)	0.050 (5)	0.062 (5)	−0.031 (5)	−0.027 (6)	0.000 (4)

### Geometric parameters (Å, °)

**Table:**

W1—C2	2.019 (10)	Zr1—C8	2.511 (9)
W1—C1^i^	2.030 (8)	O2—C2	1.139 (11)
W1—C1	2.030 (7)	O3—C3	1.134 (8)
W1—C3^i^	2.033 (7)	O4—C4	1.270 (10)
W1—C3	2.033 (7)	C4—C5	1.480 (14)
W1—C4	2.192 (9)	C5—H5A	0.9800
Cl1—Zr1	2.441 (3)	C5—H5B	0.9800
O1—C1	1.131 (9)	C5—H5C	0.9800
Zr1—O4	2.026 (6)	C6—C7	1.390 (13)
Zr1—C9	2.482 (8)	C6—C10	1.391 (13)
Zr1—C9^i^	2.482 (8)	C6—H6	0.9500
Zr1—C10	2.485 (8)	C7—C8	1.373 (13)
Zr1—C10^i^	2.485 (8)	C7—H7	0.9500
Zr1—C6	2.496 (8)	C8—C9	1.387 (13)
Zr1—C6^i^	2.496 (8)	C8—H8	0.9500
Zr1—C7^i^	2.506 (8)	C9—C10	1.362 (12)
Zr1—C7	2.506 (8)	C9—H9	0.9500
Zr1—C8^i^	2.511 (9)	C10—H11	0.9500
			
C2—W1—C1^i^	92.2 (3)	Cl1—Zr1—C8^i^	80.2 (3)
C2—W1—C1	92.2 (3)	C9—Zr1—C8^i^	108.8 (3)
C1^i^—W1—C1	85.8 (5)	C9^i^—Zr1—C8^i^	32.3 (3)
C2—W1—C3^i^	90.7 (3)	C10—Zr1—C8^i^	120.3 (3)
C1^i^—W1—C3^i^	90.4 (3)	C10^i^—Zr1—C8^i^	53.0 (3)
C1—W1—C3^i^	175.3 (3)	C6—Zr1—C8^i^	151.7 (4)
C2—W1—C3	90.7 (3)	C6^i^—Zr1—C8^i^	52.7 (3)
C1^i^—W1—C3	175.3 (3)	C7^i^—Zr1—C8^i^	31.8 (3)
C1—W1—C3	90.4 (3)	C7—Zr1—C8^i^	157.4 (5)
C3^i^—W1—C3	93.2 (4)	O4—Zr1—C8	116.0 (3)
C2—W1—C4	176.9 (4)	Cl1—Zr1—C8	80.2 (3)
C1^i^—W1—C4	90.1 (3)	C9—Zr1—C8	32.3 (3)
C1—W1—C4	90.1 (3)	C9^i^—Zr1—C8	108.8 (3)
C3^i^—W1—C4	87.2 (3)	C10—Zr1—C8	53.0 (3)
C3—W1—C4	87.2 (3)	C10^i^—Zr1—C8	120.3 (3)
O1—C1—W1	178.5 (8)	C6—Zr1—C8	52.7 (3)
O4—Zr1—Cl1	95.6 (2)	C6^i^—Zr1—C8	151.7 (4)
O4—Zr1—C9	133.5 (2)	C7^i^—Zr1—C8	157.4 (5)
Cl1—Zr1—C9	104.0 (3)	C7—Zr1—C8	31.8 (3)
O4—Zr1—C9^i^	133.5 (2)	C8^i^—Zr1—C8	125.7 (5)
Cl1—Zr1—C9^i^	104.0 (3)	O2—C2—W1	178.1 (9)
C9—Zr1—C9^i^	82.1 (4)	O3—C3—W1	178.4 (7)
O4—Zr1—C10	108.7 (3)	C4—O4—Zr1	177.4 (7)
Cl1—Zr1—C10	132.9 (2)	O4—C4—C5	112.3 (9)
C9—Zr1—C10	31.8 (3)	O4—C4—W1	123.0 (7)
C9^i^—Zr1—C10	88.1 (3)	C5—C4—W1	124.7 (7)
O4—Zr1—C10^i^	108.7 (3)	C4—C5—H5A	109.5
Cl1—Zr1—C10^i^	132.9 (2)	C4—C5—H5B	109.5
C9—Zr1—C10^i^	88.1 (3)	H5A—C5—H5B	109.5
C9^i^—Zr1—C10^i^	31.8 (3)	C4—C5—H5C	109.5
C10—Zr1—C10^i^	76.8 (4)	H5A—C5—H5C	109.5
O4—Zr1—C6	80.8 (3)	H5B—C5—H5C	109.5
Cl1—Zr1—C6	122.6 (3)	C7—C6—C10	108.2 (9)
C9—Zr1—C6	53.0 (3)	C7—C6—Zr1	74.2 (5)
C9^i^—Zr1—C6	119.7 (4)	C10—C6—Zr1	73.4 (5)
C10—Zr1—C6	32.4 (3)	C7—C6—H6	125.9
C10^i^—Zr1—C6	101.2 (4)	C10—C6—H6	125.9
O4—Zr1—C6^i^	80.8 (3)	Zr1—C6—H6	118.4
Cl1—Zr1—C6^i^	122.6 (3)	C8—C7—C6	107.2 (9)
C9—Zr1—C6^i^	119.7 (4)	C8—C7—Zr1	74.3 (5)
C9^i^—Zr1—C6^i^	53.0 (3)	C6—C7—Zr1	73.5 (5)
C10—Zr1—C6^i^	101.2 (4)	C8—C7—H7	126.4
C10^i^—Zr1—C6^i^	32.4 (3)	C6—C7—H7	126.4
C6—Zr1—C6^i^	113.3 (6)	Zr1—C7—H7	117.8
O4—Zr1—C7^i^	85.2 (3)	C7—C8—C9	108.4 (10)
Cl1—Zr1—C7^i^	90.5 (3)	C7—C8—Zr1	73.9 (5)
C9—Zr1—C7^i^	135.4 (3)	C9—C8—Zr1	72.7 (5)
C9^i^—Zr1—C7^i^	53.4 (3)	C7—C8—H8	125.8
C10—Zr1—C7^i^	130.2 (3)	C9—C8—H8	125.8
C10^i^—Zr1—C7^i^	53.7 (3)	Zr1—C8—H8	119.4
C6—Zr1—C7^i^	145.0 (5)	C10—C9—C8	108.4 (9)
C6^i^—Zr1—C7^i^	32.3 (3)	C10—C9—Zr1	74.2 (5)
O4—Zr1—C7	85.2 (3)	C8—C9—Zr1	75.0 (5)
Cl1—Zr1—C7	90.5 (3)	C10—C9—H9	125.8
C9—Zr1—C7	53.4 (3)	C8—C9—H9	125.8
C9^i^—Zr1—C7	135.4 (3)	Zr1—C9—H9	116.9
C10—Zr1—C7	53.7 (3)	C9—C10—C6	107.7 (8)
C10^i^—Zr1—C7	130.2 (3)	C9—C10—Zr1	73.9 (5)
C6—Zr1—C7	32.3 (3)	C6—C10—Zr1	74.2 (5)
C6^i^—Zr1—C7	145.0 (5)	C9—C10—H11	126.2
C7^i^—Zr1—C7	170.5 (6)	C6—C10—H11	126.2
O4—Zr1—C8^i^	116.0 (3)	Zr1—C10—H11	117.7

Symmetry codes: (i) *x*, −*y*+1/2, *z*.
